# Bulimia nervosa severity levels based on shape/weight overvaluation explain more variance in clinical characteristics than DSM-5 severity levels

**DOI:** 10.1017/S0033291725100597

**Published:** 2025-06-30

**Authors:** Sophie R. Abber, Marley G. Billman Miller, Antonia Hamilton, Shelby N. Ortiz, Ross C. Jacobucci, Jamal H. Essayli, Thomas E. Joiner, April R. Smith, Lauren N. Forrest

**Affiliations:** 1 Department of Psychology, Florida State University, Tallahassee, FL, USA; 2 Department of Psychological Sciences, Auburn University, Auburn, AL, USA; 3 Department of Psychology, Syracuse University, Syracuse, NY, USA; 4 Department of Psychiatry, University of North Carolina at Chapel Hill, Chapel Hill, NC, USA; 5 Department of Psychology, University of Notre Dame, Notre Dame, IN, USA; 6 Department of Adolescent Medicine, Penn State College of Medicine, Hershey, PA, USA; 7 Department of Psychology, University of Oregon, Eugene, OR, USA

**Keywords:** bulimia nervosa, classification, exploratory data mining, SEM Trees, severity, eating disorders

## Abstract

**Background:**

*DSM-5* specifies bulimia nervosa (BN) severity based on specific thresholds of compensatory behavior frequency. There is limited empirical support for such severity groupings. Limited support could be because the *DSM-5*’s compensatory behavior frequency cutpoints are inaccurate or because compensatory behavior frequency does not capture true underlying differences in severity. In support of the latter possibility, some work has suggested shape/weight overvaluation or use of single versus multiple purging methods may be better severity indicators. We used structural equation modeling (SEM) Trees to empirically determine the ideal variables and cutpoints for differentiating BN severity, and compared the SEM Tree groupings to alternate severity classifiers: the *DSM-5* indicators, single versus multiple purging methods, and a binary indicator of shape/weight overvaluation.

**Methods:**

Treatment-seeking adolescents and adults with BN (*N* = 1017) completed self-report measures assessing BN and comorbid symptoms. SEM Trees specified an outcome model of BN severity and recursively partitioned this model into subgroups based on shape/weight overvaluation and compensatory behaviors. We then compared groups on clinical characteristics (eating disorder symptoms, depression, anxiety, and binge eating frequency).

**Results:**

SEM Tree analyses resulted in five severity subgroups, all based on shape/weight overvaluation: overvaluation <1.25; overvaluation 1.25–3.74; overvaluation 3.75–4.74; overvaluation 4.75–5.74; and overvaluation ≥5.75. SEM Tree groups explained 1.63–6.41 times the variance explained by other severity schemes.

**Conclusions:**

Shape/weight overvaluation outperformed the *DSM-5* severity scheme and single versus multiple purging methods, suggesting the *DSM-5* severity scheme should be reevaluated. Future research should examine the predictive utility of this severity scheme.

## Introduction

Bulimia nervosa (BN) is an eating disorder (ED) characterized by recurrent binge-eating episodes and compensatory behaviors (e.g., self-induced vomiting, maladaptive exercise) and overvaluation of body shape/weight. The revised fifth edition of the *Diagnostic and Statistical Manual of Mental Disorders* (*DSM-5-TR;* APA, 2022 includes severity specifiers for BN based on compensatory behavior frequency, defined as: mild (1–3 episodes/week), moderate (4–7 episodes/week), severe (8–13 episodes/week), and extreme (≥14 episodes/week). While these severity levels ideally would suggest intervention targets and represent the degree of functional impairment, the *DSM-5-TR* severity scheme was not empirically validated. Thus, it is unknown: (1) whether compensatory behavior frequency is the most appropriate metric by which to define BN severity and (2) what are the precise levels of compensatory behaviors or a more appropriate metric that most accurately differentiates BN severity groups.

A systematic review and meta-analysis found that compensatory behavior frequency provides some clinical utility as a severity specifier for BN but also found support for alternative severity rating schemes (weight/shape overvaluation and drive for thinness) (Dang, Giles, Fuller-Tyszkiewicz, Kiropoulos, & Krug, 2022). While Dang et al. (2022) found some support for the *DSM-5* BN severity specifiers, their review and meta-analysis focused on ED psychopathology as a validator of the specifiers, and other variables across domains (e.g., general psychopathology, quality of life) may also be important in validating BN severity specifiers. Some other work has examined the validity and utility of the current *DSM-5* BN severity specifiers by assessing whether *DSM-5*-defined severity groups differed on depression, anxiety, quality of life, and physical health. To our knowledge, three studies have compared BN severity groups on depression, with two finding no differences (Gianini et al., 2017; Smith et al., 2017) and one finding differences between groups (Grilo, Ivezaj, & White, 2015). Additionally, Smith et al. (2017) found that *DSM*-*5*-defined BN severity levels differed on anxiety and quality of life but not physical health. Taken together, the validity of the BN specifiers in their current form is still unclear.

Alternative variables to classify ED severity have been explored, including use of multiple purging methods for BN (Gianini et al., 2017), drive for thinness (Krug et al., 2021), and shape/weight overvaluation in anorexia nervosa (Billman Miller et al., 2025), binge-eating disorder (Forrest, Jacobucci, & Grilo, 2022), and transdiagnostically (Gianini et al., 2017). Gianini et al. (2017) explored the number of purging methods, rather than the frequency of purging behaviors, to classify severity. They found that defining severity based on the number of purging methods was more strongly associated with psychopathology than compensatory behavior frequency. Krug et al. (2021) found that drive for thinness may be a useful transdiagnostic severity specifier with greater clinical utility than compensatory behavior frequency. Gianini et al. (2017) explored clinically significant shape/weight overvaluation as a severity grouping, and found that this severity scheme outperformed the *DSM*-based severity specifiers. Similarly, Jenkins, Luck, Cardy, and Staniford (2016) found that classification of BN severity based on the *DSM-5* indicators (frequency of compensatory behaviors) was more accurate when considered in the presence of shape/weight overvaluation.

Shape/weight overvaluation may be a valid, clinically useful metric of severity for BN. Importantly, shape/weight overvaluation can take on different meanings in different contexts, as it can be considered a symptom of BN or a mechanism specific to enhanced cognitive behavior therapy for EDs (Cooper & Fairburn, 2010). In the context of this study, we refer to shape/weight overvaluation as a theory-agnostic representation of the DSM-5’s BN criterion D (“self-evaluation is unduly influenced by body shape and weight,” APA, 2022). Prior work (Forrest, Jones, Ortiz, & Smith, 2018; Gianini et al., 2017; Grilo et al., 2015; Ojserkis, Sysko, Goldfein, & Devlin, 2012) evaluating the utility of shape/weight overvaluation as an alternative severity specifier has relied on a binary scheme, where individuals either do or do not experience clinically significant overvaluation. Given that shape/weight overvaluation is a continuous construct, it is possible that there may be more than one meaningful overvaluation cutpoint that differentiates BN severity. Thus, exploring shape/weight overvaluation beyond a binary operationalization is critical to advance our understanding of this symptom as a potential metric of BN severity.

Structural equation modeling trees (SEM Trees), which empirically determine specific thresholds of continuous variables that best differentiate groups (Brandmaier, von Oertzen, McArdle, & Lindenberger, 2013), may be particularly useful in answering outstanding questions about what symptoms best reflect BN severity and what cutpoint levels are most valid. SEM Trees can accommodate more than one variable on which to determine cutpoints (Brandmaier et al., 2013), allowing for comparison of how different variables contribute to groupings (e.g., compensatory behavior frequency and shape/weight overvaluation). SEM Trees contain confirmatory and exploratory components, where a confirmatory *outcome model* is based on theory or evidence; an exploratory *decision tree* uses *covariates* to split the data into subgroups that best fit the outcome model (Brandmaier et al., 2013). SEM Trees have been used to empirically determine ED severity for patients with binge-eating disorder (Forrest et al., 2022), other specified feeding or eating disorder (Ortiz, Forrest, Kinkel-Ram, Jacobucci, & Smith, 2021), and anorexia nervosa (Billman Miller et al., 2025). These studies modeled a latent ED severity variable comprising ED symptoms and comorbid symptoms (e.g., depression symptoms, anxiety symptoms, number of psychiatric comorbidities) and used SEM Trees to identify whether shape/weight overvaluation was an appropriate (and/or superior to existing *DSM-5* severity specifiers, for AN and BED) metric of severity. Past work (Billman Miller et al., 2025; Forrest et al., 2022; Ortiz et al., 2021) has included comorbid symptoms as part of the latent outcome model given high comorbidity between AN, BED, and OSFED and depression and anxiety symptoms. Similarly, there is high comorbidity between these symptoms and BN in both adolescent (Herpertz-Dahlmann, 2015) and adult (Bulik, 2002; Godart et al., 2007) populations. Across studies, two key findings have been observed: (1) SEM Trees identified multiple severity subgroups defined by increasing levels of (continuously modeled) shape/weight overvaluation (though see Ortiz et al., 2021, where only two severity subgroups were identified) and (2) SEM Tree groupings explained approximately 2 times and 20 times more variance in clinical characteristics (ED and comorbid symptoms) than *DSM-5* severity groupings in BED and AN, respectively. No studies to our knowledge have utilized SEM Trees or other data-driven methods to empirically identify severity for BN.

Thus, we had three overarching aims: (1) determine optimal cutpoint values for severity levels in BN using compensatory behavior frequency and/or shape/weight overvaluation; (2) compare whether compensatory behavior frequency or shape/weight overvaluation accounted for more severity cutpoints in BN; and (3) compare the extent to which SEM Tree-defined severity groups, *DSM*-5 severity groups, and alternative severity groupings (clinically significant shape/weight overvaluation, >1 purging method) differentiate people with BN based on clinical characteristics. We created a latent outcome model of BN severity based on ED, depression, and anxiety symptoms, consistent with prior work. We did not have specific hypotheses for the exact levels of compensatory behaviors and/or shape/weight overvaluation that would differentiate groups, as analyses were exploratory in nature. We expected, based on prior work, that shape/weight overvaluation levels and the use of multiple purging methods would outperform the *DSM-5* severity indicators based on compensatory behavior frequency.

## Method

### Participants

Participants (76% White, 99% women, *M*_age_ = 24.71, SD_age_ = 9.22; Table 1) receiving treatment for BN in residential (*n* = 770), partial hospitalization program (PHP; *n* = 192), and outpatient care (*n* = 55) were recruited from 2014 to 2021. The residential and PHP centers provided treatment only to women and girls, while all genders were served in the outpatient center. Data were collected from two residential sites, 20 PHP sites, and one outpatient site. Some participants were transferred between levels of care. When participants were present in more than one dataset, data were used for only their first admission. Data were combined across levels of care to increase the range of ED severity in our sample.

**Table 1.** Gender, race, and age distributions for SEM Tree-derived groups

	All groups *n* = 1017	1 *n* = 34	2 *n* = 109	3 *n =* 111	4 *n* = 177	5 *n* = 582		
	*n* (%)	*n* (%)	*n* (%)	*n* (%)	*n* (%)	*n* (%)	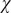 ^2^	*p*
Gender							13.90	.31
Cis man	3 (0.01%)	1 (3%)	1 (1%)	0 (0%)	0 (0%)	1 (0.01%)		
Cis woman	1,006 (99%)	33 (97%)	108 (99%)	110 (99%)	176 (99%)	579 (99%)		
Trans man	3 (0.01%)	0 (0%)	0 (0%)	1 (1%)	1 (1%)	1 (0.01%)		
Trans woman	0 (0%)	0 (0%)	0 (0%)	0 (0%)	0 (0%)	0 (0%)		
Gender diverse	1 (0.01%)	0 (0%)	0 (0%)	0 (0%)	0 (0%)	1 (0.01%)		
Race							21.58	.60
American Indian or Alaska Native	8 (1%)	0 (0%)	2 (2%)	2 (2%)	1 (1%)	3 (1%)		
Asian	36 (4%)	2 (6%)	8 (7%)	3 (3%)	8 (5%)	15 (3%)		
Black	30 (3%)	1 (3%)	2 (2%)	2 (2%)	6 (4%)	19 (3%)		
Hispanic	71 (7%)	0 (0%)	6 (6%)	7 (6%)	16 (9%)	42 (7%)		
Multiracial	66 (6%)	4 (13%)	6 (6%)	8 (7%)	10 (6%)	38 (7%)		
White	771 (76%)	25 (75%)	79 (73%)	84 (76%)	129 (75%)	454 (79%)		
Not reported	8 (1%)	0 (0%)	1 (1%)	2 (2%)	2 (1%)	3 (1%)		
	*M* (*SD*)	*M* (*SD*)	*M* (*SD*)	*M* (*SD*)	*M* (*SD*)	*M* (*SD*)	*F*	*p*
Age	24.72 (9.24)	24.35 (9.84)	25.63 (9.60)	25.68 (8.96)	23.21 (8.44)	24.85 (9.39)	1.79	.13

Our initial sample size was *N* = 1064. Participants were excluded if: (1) they had missing data on all measures (*n* = 31); (2) their EDE-Q Global score was <0.5 (*n* = 10) and they were in residential or PHP treatment, given that this is an indicator of invalid responding at these levels of care (Thompson-Brenner et al., 2019); or (3) reported no core diagnostic indicators of BN on the EDE-Q or had missing data for all of these items (*n* = 6; see Supplemental Material). These exclusions resulted in an analytic sample of *n* = 1017.

### Procedures

Participants completed self-reports assessing cognitive ED, anxiety, and depression symptoms prior to beginning treatment. BN diagnoses were assigned at intake via unstructured clinical interview by a master’s level clinician and confirmed by the staff psychiatrist at admission. Interrater reliability data were not available. All participants provided informed consent for their data to be used for research and procedures were approved by the Pennsylvania State University Institutional Review Board (#00019147) as nonhuman subjects research due to the present analyses being secondary data analysis.

### Measures

#### Outcome model: latent BN severity

Latent BN severity was modeled using cognitive BN symptoms, depression symptoms, and anxiety symptoms, where all factor loadings were constrained to equally represent the latent factor. We tested a model with unequal factor loadings, but estimation problems occurred. Depression and anxiety symptoms were selected as indicators given high comorbidity between these symptoms and BN in both adolescent (Herpertz-Dahlmann, 2015) and adult (Bulik, 2002; Godart et al., 2007) populations, suggesting that BN is unlikely to occur in isolation without depression or anxiety. Further, research suggests anxiety and depression are related to higher levels of ED symptom severity and poorer outcome (Sander, Moessner, & Bauer, 2021).

**Cognitive ED symptoms**. We assessed cognitive ED symptoms using 22 items from the EDE-Q (Fairburn & Beglin, 2008). Items are scored on a 0- to 6-point Likert scale and combined into a composite Global score (range: 0–6), with higher scores reflecting more severe symptoms. We calculated Global scores *without* the overvaluation items to minimize overlap between the variables by which severity may be defined (overvaluation) and the variables on which severity subgroups would be compared (EDE-Q Global score). Internal consistency was excellent across samples (*αs* = .92–.94).

**Depression symptoms.** We assessed depression symptoms using the Center for Epidemiological Studies Depression Scale (CESD, used in the residential and PHP samples; (Radloff, 1977) or the Patient Health Questionnaire-9 (PHQ-9, used in the outpatient sample; (Kroenke, Spitzer, & Williams, 2001). The CESD comprises 20 items on a 0 (rarely or none of the time) to 3 (most or almost all the time) point Likert scale. Items are summed to a total score (range: 0–60), with higher scores representing more severe depression. The PHQ-9 comprises 9 items on a Likert scale ranging from 0 (not at all) to 3 (nearly every day). Items are summed to a total score (range: 0–27), with higher scores representing more severe depression. Internal consistency was excellent on the CESD (*αs* = .90). We were unable to calculate internal consistency for the PHQ-9, as itemized data were not available.

**Anxiety symptoms.** We assessed anxiety symptoms using the Overall Anxiety Severity and Intensity Scale (OASIS, used in the residential and PHP samples; (Campbell-Sills et al., 2009) or the Generalized Anxiety Disorder-7 (GAD-7, used in the outpatient sample; (Spitzer, Kroenke, Williams, & Löwe, 2006). The OASIS comprises five items on a 0–4 point Likert scale. Items are summed to a total score (range: 0–20), with higher scores representing higher anxiety. The GAD-7 comprises seven items on a Likert scale ranging from 0 to 4. Items are summed to a total score (range: 0–28). Internal consistency was excellent on the OASIS (*αs* = .86–.87). We were unable to calculate internal consistency for the GAD-7, as itemized data were not available.

#### Model covariates

We included two covariates (i.e., the variables used to partition data into subgroups) in the model: severity of shape/weight overvaluation and compensatory behavior frequency. Compensatory behavior frequency was defined broadly as self-induced vomiting, laxative or diuretic use, and excessive exercise. Shape/weight overvaluation was assessed using a composite of two strongly correlated EDE-Q items (“Has your weight influenced how you think about (judge) yourself as a person?” and “Has your shape influenced how you think about (judge) yourself as a person?”; *r* = 0.80, *p* < .001*).* Compensatory behavior frequency was the sum of the EDE-Q items assessing frequency of self-induced vomiting, laxative or diuretic use, and excessive exercise. To control for outliers, each behavioral frequency item was capped at 56 prior to summing into the composite variable, as this translates to 2 instances per day for 28 days (i.e., the closest approximation to the *DSM-5* definition of extreme BN severity).

#### Statistical analysis

We *z*-scored all outcome model indicators so that constructs assessed using different measures (e.g., depression was assessed in the residential and PHP samples using the CESD but in the outpatient sample using the PHQ-9) could be combined into a single score on a common scale. Although cognitive ED symptoms were assessed using the same measure across samples, we also *z*-scored EDE-Q Global scores (within each level of care), so that all outcome model indicators had a common scale.

**Figure 1.**
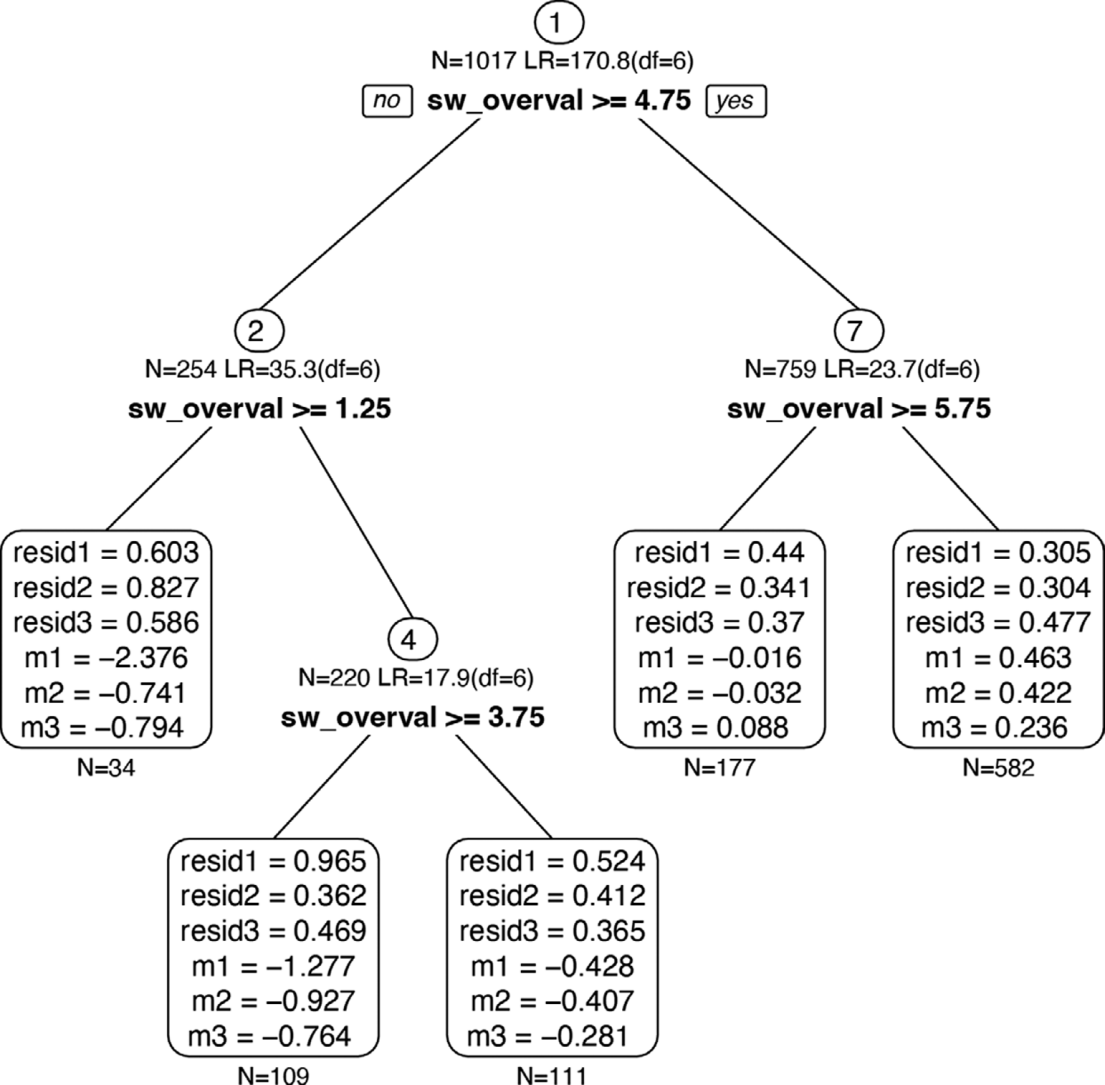
Decision tree with splits based on shape/weight overvaluation and compensatory behaviors. *Note.* LR = likelihood ratio; resid1 = residual variance of cognitive ED symptoms; resid2 = residual variance of depression, resid3 = residual variance of anxiety. m1 = manifest mean of cognitive ED symptoms, m2 = manifest mean of depression, m3 = manifest mean of anxiety. Manifest means are the means of each variable used in the latent outcome model. Residual variance is unexplained variance in indicators that are not explained by the latent BN severity variable.

**SEM Trees.** Since there were only three indicators in the confirmatory BN severity outcome model, model fit of the latent severity variable could not be assessed. The exploratory decision tree recursively separated data into subgroups that explained the maximum variance in the outcome model; groups were based on values (splits) of the covariates: shape/weight overvaluation and compensatory behavior frequency. We used a ‘fair’ splitting criterion (Brandmaier et al., 2013), where the sample is randomly divided in two equal parts, to control for the number of response options in the covariates. The outcome model is compared at every possible value of the covariates in the first part, and the value resulting in the largest model fit improvement is selected. That split value is then evaluated in the second part of the sample. A retained split indicates a cutpoint that differentiates severity subgroups (see Figure 1). Given that SEM Trees accommodate multiple covariates, we included both compensatory behavior frequency and shape/weight overvaluation in the same model, as this allows us to directly compare each variable’s contribution to model fit.

**SEM forests.** SEM Trees are singular trees with inherent variability that could drive splits and therefore bias conclusions regarding which covariate is the superior indicator of BN severity. SEM Forests can mitigate this issue by estimating multiple individual SEM Trees and evaluating within a “forest” of trees which variable is a better overall indicator of severity. We used SEM Forests to estimate 100 individual trees and compared one variable per split (Brandmaier, Prindle, McArdle, & Lindenberger, 2016). The forest derives an importance parameter for each covariate, indicating the relative strength of shape/weight overvaluation versus compensatory behavior frequency in improving model fit.

**Planned comparisons.** We used ANOVAs with orthogonal, planned contrasts comparing lower SEM Tree severity groups to higher SEM Tree severity groups (e.g., Group 1 versus Groups 2–5) on clinical characteristics. Partial eta squared was used to indicate overall effect sizes. Cohen’s *d* was calculated to index contrast effect sizes.

In addition to the SEM Forests described above to test whether compensatory behavior frequency or shape/weight overvaluation was the superior metric of severity within our SEM Tree model, we assessed whether SEM Tree groups outperformed existing severity indicators by comparing clinical characteristics using three other severity subgrouping schemes: (1) *DSM-5* levels; (2) clinically significant shape/weight overvaluation (either item rated as ≥4); and (3) single versus multiple purging methods. These comparisons were made using ANOVAs with orthogonal, planned contrasts, and *t* tests. Effect sizes were calculated for all comparisons. For each severity subgrouping scheme, we did not compare clinical characteristics that were used to define severity subgroups (e.g., did not compare compensatory behavior frequency across DSM-5 groups given that *DSM-5* groups are defined by compensatory behavior frequency). Effect sizes were also descriptively compared for each severity classification scheme.

**Normality, assumptions, and missing data.** We inspected normality for all variables. Levene’s test was used to determine whether equal variances could be assumed. We report Welch-corrected ANOVAs and contrasts where indicated.

The maximum amount of missingness for all measures except the anxiety variable was 5.0%. The anxiety variable was missing 35% of responses. Much of the missingness (97%) is a result of the PHP and residential treatment facilities not adding in the OASIS to the intake battery until approximately 2 years after they began administering the EDE-Q and CESD. Participants admitted before versus after the inclusion of the OASIS did not differ on EDE-Q global scores or shape/weight overvaluation, although they did report higher depression and greater compensatory behavior frequency (Supplemental Table 2). However, all means were within 1 SD of one another, and effect sizes were small or small–moderate. Missing data for the outcome model were handled with full information maximum likelihood. Missing data for the comparisons among severity subgroups were handled using pairwise deletion.

## Results

**SEM tree and SEM forest.** Four splits were identified, creating five severity subgroups (Figure 1). Group 1 was people with overvaluation <1.25 (*n* = 34). Group 2 was people with overvaluation 1.25–3.74 (*n* = 109). Group 3 was people with overvaluation 3.75–4.74 (*n* = 111). Group 4 was people with overvaluation 4.75–5.74 (*n* = 177). Group 5 was people with overvaluation ≥5.75 (*n* = 582). No splits occurred at any values of compensatory behaviors. SEM Forests found overvaluation resulted in 263.4 units of improvement in model fit (−2 log likelihood), whereas compensatory behavior frequency resulted in only 23.5 units of improvement in model fit.

ANOVAs showed SEM Tree groups differed on ED symptoms, depression, anxiety, and binge-eating frequency with medium-to-large effect sizes (Table 2; all *p*s < .01). Planned contrasts (Table 3) indicated that cognitive ED symptoms and depression increased as the level of overvaluation increased (all *p*s < .001). Exceptions to this pattern were found for anxiety and binge-eating frequency. Although anxiety was different for SEM Tree contrasts 1, 2, and 3, where lower severity groups had lower anxiety than higher severity groups (all *p*s < .001), anxiety did not differ between SEM Tree Groups 4 versus 5 (*p* = .34). No contrasts were significant for binge-eating frequency (all *p*s > .05).

**Table 2.** Clinical characteristics compared among the structural equation model tree-derived groups

	1 *n* = 34	2 *n* = 109	3 *n =* 111	4 *n* = 177	5 *n* = 582			
	*n* (%)	*n* (%)	*n* (%)	*n* (%)	*n* (%)	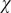 ^2^	p	
Level of care						9.20	.33	
Outpatient	2 (3.7)	6 (11.1)	7 (13.0)	12 (22.2)	27 (50.0)			
PHP	3 (1.6)	19 (9.9)	30 (15.7)	32 (16.8)	107 (56.0)			
Residential	29 (3.8)	84 (10.9)	74 (9.6)	133 (17.3)	448 (58.3)			
	*M* (*SD*)	*M* (*SD*)	*M* (*SD*)	*M* (*SD*)	*M* (*SD*)	*F*	*p*	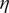 ^2^ (95% CI)
Overvaluation^b^	0.66 (0.46)	2.65 (0.67)	4.17 (0.24)	5.16 (0.24)	6.00 (0.00)	–	–	–
Compensatory behavior frequency	4.53 (3.89)	5.31 (4.29)	6.22 (5.13)	7.33 (4.96)	7.54 (5.79)	6672.62	<.001	0.96 (0.96, 0.97)
Cognitive ED symptoms z-score^a^	−2.38 (0.64)	−1.28 (0.97)	−0.43 (0.76)	−0.02 (0.70)	0.46 (0.63)	242.34	<.001	0.51 (0.47, 0.58)
Depression z-score^a^	−0.74 (1.18)	−0.93 (1.01)	−0.40 (1.00)	−0.03 (0.87)	0.42 (0.81)	61.41	<.001	0.23 (0.18, 0.27)
Anxiety z-score	−0.94 (1.10)	−0.75 (0.99)	−0.27 (1.01)	0.11 (0.87)	0.20 (0.93)	23.46	<.001	0.12 (0.08, 0.16)
Binge eating frequency	3.35 (3.11)	3.40 (3.03)	3.56 (2.90)	3.93 (3.64)	4.41 (3.61)	3.40	<.01	0.01 (0.00, 0.03)

a Unequal variances across groups;

b Presented for descriptive purposes. We did not statistically compare overvaluation across groups, as this was the metric by which groups were empirically defined. For the level of care, percentages indicate the percent within each level of care within each severity group (i.e., rows sum to 100). *n* differs for anxiety as a result of missing data. Binge-eating frequency = weekly binge-eating frequency; ED = eating disorder.

**Table 3.** Structural equation model tree-derived groups’ contrast results

	1 versus 2 + 3 + 4 + 5			2 versus 3 + 4 + 5			3 versus 4 + 5			4 versus 5		
	*t* (*df*)	*p*	*d* (95% CI)	*t* (*df*)	*p*	*d* (95% CI)	*t* (*df*)	*p*	*d* (95% CI)	*t* (*df*)	*p*	*d* (95% CI)
Cognitive ED symptoms z-score^a^	17.95 (39.07)	< .001	11.80 (10.33, 13.27)	13.17 (132.88)	< .001	5.50 (4.83, 6.16)	8.37 (147.21)	< .001	1.98 (1.56, 2.39)	8.21 (268.92)	< .001	0.74 (0.57, 0.92)
Depression z-score^a^	2.45 (35.45)	.02	2.30 (0.91, 3.68)	8.80 (147.24)	< .001	3.19 (2.55, 3.82)	5.79 (143.44)	< .001	1.40 (0.99, 1.82)	6.07 (262.49)	< .001	0.55 (0.38, 0.72)
Anxiety z-score	3.47 (688)	< .001	3.25 (1.40, 5.10)	6.46 (688)	< .001	2.44 (1.69, 3.18)	3.58 (688)	<.001	0.91 (0.42, 1.41)	0.97 (688)	.34	0.10 (−0.10, 0.31)
Binge eating frequency	0.78 (997)	.43	0.56 (−0.83, 1.94)	1.56 (997)	.12	0.49 (−0.13, 1.11)	1.66 (997)	.10	0.34 (−0.07, 0.76)	1.60 (997)	.11	0.13 (−0.04, 0.30

*Note.* Binge-eating frequency = weekly binge-eating frequency; ED = eating disorder.

a Significant heterogeneity of variance and thus Welch-corrected contrasts are reported. Comparisons are not presented for overvaluation, as overvaluation was the primary metric by which SEM Tree groups were defined.

***DSM-5* severity specifiers.** ANOVAs indicated *DSM-5* groups differed on overvaluation, cognitive ED symptoms, depression, and binge-eating frequency (all *p*s < .001) with medium effect sizes but did not differ on anxiety (Table 4; *p* = .19). As expected, contrasts (Table 5) indicated all *DSM-5* groups differed on binge-eating frequency, such that increased *DSM-5* severity grouping was associated with greater binge-eating frequency (all ps < .001). Moderate through extreme groups had higher shape/weight overvaluation, cognitive ED symptoms, and depression than the mild group (all *p*s < .001) but similar levels of anxiety (*p* = .15). Severe and extreme groups had higher shape/weight overvaluation, cognitive ED symptoms, and depression than the moderate group (all *p*s < .05) but similar levels of anxiety (*p* = .55). Severe and extreme groups did not differ on any symptoms (all *p*s > .05).

**Table 4.** Group comparisons of demographic and clinical characteristics for *Diagnostic and Statistical Manual for Mental Disorders-5*-specified severity indicators.

	Mild *n* = 462	Moderate *n* = 182	Severe *n* = 226	Extreme *n* = 139			
	*n* (%) or *M* (*SD*)	*n* (%) or *M* (*SD*)	*n* (%) or *M* (*SD*)	*n* (%) or *M* (*SD*)	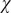 ^2^ or *F*	*p*	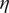 ^2^ (90% CI)
Level of care					22.64	< .001	–
Outpatient	26 (54.2)	7 (14.6)	13 (27.1)	2 (4.2)			
PHP	111 (57.8)	37 (19.3)	32 (16.7)	12 (6.3)			
Residential	325 (42.3)	138 (18.0)	181 (23.5)	125 (16.3)			
Overvaluation	4.98 (1.51)	4.91 (1.48)	5.31 (1.21)	5.53 (0.95)	11.75	< .001	0.03 (0.02, 0.05)
Compensatory behavior frequency^a^	3.02 (2.73)	5.45 (0.83)	10.44 (1.73)	16.78 (3.77)	–	–	–
Cognitive ED symptoms z-score^b^	−0.23 (1.07)	−0.07 (0.98)	0.29 (0.83)	0.39 (0.76)	26.84	< .001	0.07 (0.04, 0.10)
Depression z-score^b^	−0.07 (1.03)	0.04 (1.00)	0.26 (0.96)	0.24 (0.86)	7.49	< .001	0.02 (0.01, 0.04)
Anxiety z-score	−0.07 (0.98)	0.00 (1.04)	0.15 (1.00)	−0.02 (1.00)	1.61	.19	0.05 (0.00, 0.02)
Binge eating frequency^b^	3.40 (3.10)	3.73 (2.52)	4.20 (3.23)	6.58 (4.82)	19.06	< .001	0.09 (0.03, 0.08)

*Note.* Comp. bx frequency = weekly compensatory behavior frequency; overvaluation = shape and weight overvaluation; binge eating = weekly binge-eating frequency; ED = eating disorder.

a Presented for descriptive purposes. We did not statistically compare compensatory behavior frequency across groups, as it was the metric by which *DSM-5* groups were defined

b Significant heterogeneity of variance and thus Welch-corrected contrasts are reported.

**Table 5.** Contrast results for *Diagnostic and Statistical Manual for Mental Disorders-5*-specified severity indicators.

	Mild versus Moderate + Severe + Extreme			Moderate versus Severe + Extreme			Severe versus Extreme		
	*t* (*df*)	*p*	*d* (95% CI)	*t* (*df*)	*p*	*d* (95% CI)	*t* (*df*)	*p*	*d* (95% CI)
Overvaluation^a^	3.08 (860.53)	.002	0.59 (0.22, 0.97)	4.09 (278.68)	< .001	0.81 (0.45, 1.17)	1.99 (339.85)	.05	0.20 (−0.01, 0.42)
ED symptoms z-score^a^	6.94 (866.47)	< .001	1.34 (0.96, 1.72)	4.92 (304.38)	< .001	0.96 (0.59, 1.32)	1.16 (312.36)	.25	0.12 (−0.09, 0.33)
Depression z-score^a^	3.98 (902.77)	< .001	0.77 (0.39, 1.15)	2.30 (327.32)	.02	0.43 (0.07, 0.80)	0.20 (313.10)	.84	−0.02 (−0.23, 0.19)
Anxiety z-score	1.45 (683)	.15	0.34 (−0.12, 0.80)	0.60 (683)	.55	0.13 (−0.31, 0.58)	−1.17 (683)	.24	−0.17 (−0.45, 0.11)
Binge eating frequency^a^	6.54 (644.54)	< .001	1.30 (0.92, 1.68)	5.57 (389.57)	< .001	0.94 (0.58, 1.31)	5.14 (214.89)	< .001	0.61 (0.39, 0.82)

*Note.* Comp. bx frequency = weekly compensatory behavior frequency; overvaluation = shape and weight overvaluation; binge eating = weekly binge-eating frequency; ED = eating disorder.

a Significant heterogeneity of variance and thus Welch-corrected contrasts are reported.

**Clinical overvaluation.** People with clinical overvaluation had higher compensatory behavior frequency, cognitive ED symptoms, depression, anxiety, and binge-eating frequency compared to those with nonclinical overvaluation (all *p*s < .01), with medium-to-large effect sizes (Table 6).

**Table 6.** Comparisons of clinical characteristics based on shape/weight overvaluation clinical threshold of 4.

	Overvaluation < 4 *n* = 142	Overvaluation ≥ 4 *n* = 863				
	*n* (%)	*n* (%)	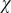 ^2^	*p*		
Level of care			1.31	.52		
Outpatient	8 (14.8)	46 (85.2)				
PHP	22 (11.5)	169 (88.5)				
Residential	113 (14.7)	655 (85.3)				
	*M* (*SD*)	*M* (*SD*)	*t* (*df*)	*p*	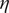 ^2^ (95% CI)	*d* (95% CI)
Overvaluation^a^	2.17 (1.05)	5.60 (0.65)	–	–	–	–
Compensatory behavior frequency	5.13 (4.20)	7.33 (5.56)	−5.50 (231.11)	< .001	.02 (0.01, 0.04)	−0.41 (−0.59, −0.23)
Cognitive ED symptoms z-score^b^	−1.54 (1.01)	0.25 (0.73)	−20.29 (167.35)	< .001	.39 (0.35, 0.43)	−2.30 (−2.50, −2.10)
Depression z-score^b^	−0.88 (1.05)	0.23 (0.90)	−11.88 (178.36)	< .001	.15 (0.12, 0.18)	−1.20 (−1.39, −1.02)
Anxiety z-score	−0.78 (1.01)	0.12 (0.94)	−8.70 (691)	< .001	.10 (0.07, 0.13)	−0.96 (−1.18, −0.74)
Binge eating frequency	3.39 (3.03)	4.21 (3.54)	−2.60 (1000)	.005	.01 (0.001, 0.02)	−0.24 (−0.41, −0.06)

*Note.* binge eating = weekly binge-eating frequency; ED = eating disorder.

a Presented for descriptive purposes. We did not statistically compare shape/weight overvaluation across groups, as it was the metric by which these groups were defined.

b Significant heterogeneity of variance and thus Welch-corrected contrasts are reported.

**Single versus multiple purging.** People who used multiple purging methods had higher compensatory behavior frequency, overvaluation, cognitive ED symptoms, and depression (all *p*s < .001) with small-to-medium effect sizes but did not differ on anxiety (*p* = .15) or binge-eating frequency (Table 7; *p* = .44).

**Table 7.** Comparisons of clinical characteristics based on single versus multiple purging methods.

	Single purging *n* = 773	Multiple purging *n* = 233				
	*n* (%) or *M* (*SD*)	*n* (%) or *M* (*SD*)	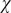 ^2^ or *t* (*df*)	*p*	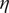 ^2^ (95% CI)	*d* (95% CI)
Level of care			5.60	.06	–	–
Outpatient	39 (81.3)	9 (18.7)				
PHP	159 (82.8)	33 (17.2)				
Residential	578 (75.2)	191 (24.8)				
Overvaluation^a^	5.02 (1.46)	5.45 (1.08)	−4.91 (509.75)	< .001	.02 (0.01, 0.03)	−0.31 (−0.46, −0.17)
Compensatory behavior frequency	5.98 (4.85)	10.48 (5.91)	−10.61 (331.14)	< .001	0.12 (0.09, 0.16)	−0.88 (−1.03, −0.73)
Cognitive ED symptoms^b^	−0.10 (1.03)	0.33 (0.78)	−6.78 (499.75)	< .001	.03 (0.01, 0.06)	−0.44 (−0.58, −0.29)
Depression^b^	−0.02 (1.02)	0.36 (0.85)	−5.63 (448.32)	< .001	.03 (0.01, 0.04)	−0.38 (−0.53, −0.24)
Anxiety	−0.04 (1.01)	0.10 (0.95)	−1.46 (685)	.15	.003 (0.00, 0.01)	−0.14 (−0.32, 0.05)
Binge eating frequency	4.12 (3.52)	3.92 (3.38)	0.77 (1004)	.44	.001 (0.00, 0.01)	0.06 (−0.09, 0.20)

*Note.* binge eating = weekly binge-eating frequency; ED = eating disorder.

a Presented for descriptive purposes. We did not statistically compare shape/weight overvaluation across groups, as it was the metric by which these groups were defined.

b Significant heterogeneity of variance and thus Welch-corrected contrasts are reported.

**Variance in clinical characteristics explained by severity schemes.** 1–51% (*M* = 21.8%) of the variance in clinical characteristics was explained by SEM Tree-derived subgroups, 2–9% (*M* = 5.2%) by *DSM-5* subgroups, 1–39% (*M* = 13.4%) by clinical overvaluation, and 0.1–12% (*M* = 3.4%) by single versus multiple purging methods. Thus, SEM Tree groups explained 4.19 times the variance explained by the *DSM*-*5* groups, 1.63 times the variance explained by the clinical overvaluation groups, and 6.41 times the variance explained by the purging method groups.

## Discussion

This study identified the compensatory behavior frequencies and/or shape/weight overvaluation levels that differentiated BN severity, determined whether compensatory behavior frequency or shape/weight overvaluation was the superior indicator of BN severity, and compared how SEM Tree-defined severity groups differentiated intensity of several clinical characteristics compared to the *DSM-5* severity grouping and alternative severity schemes. The SEM Tree identified five levels of severity, all based on shape/weight overvaluation. Since the overvaluation composite was an average of two items, all individual participant composite scores were necessarily between 0 and 6 with 0.5-level increments possible. Thus, since not all cutpoints are possible values for individual participants, we round these numbers from this point forward to facilitate the practical implications of these findings. The first split was between people with overvaluation composite scores of 1 or less. The second split was between people with overvaluation composite scores between 1.5 and 3.5. The third split was between people with overvaluation composite scores between 4 and 4.5. The fourth split was people with overvaluation composite scores above 5. The highest-severity group was the largest in size (57%), which could mean that this group is the most common clinical presentation or may reflect that most participants were receiving treatment at a higher level of care. Within the SEM Forest, shape/weight overvaluation contributed to more improvement in model fit (263.4 units) than compensatory behavior frequency (23.5 units). When examining group differences, SEM Tree-defined severity groups explained 4.2 times the variance in clinical characteristics explained by DSM-5-defined groups, 1.6 times the variance explained by clinical overvaluation groups, and 6.4 times the variance explained by purging method groups. Altogether, findings indicate that relative to other currently proposed BN severity specification schemes, shape/weight overvaluation – when modeled beyond a binary operationalization – is the strongest marker of BN severity. While further investigation of the identified overvaluation cutpoints is warranted, our findings call for reconsideration of the current *DSM-5* classification scheme.

Our results are consistent with prior work finding limited support for the *DSM-5*’s BN severity specifiers (Gianini et al., 2017; Gorrell et al., 2019; Grilo et al., 2015) and with broader findings in the field that data-driven judgments outperform clinical or expert judgments (Dawes, Faust, & Meehl, 1993; Meehl, 1954). Although *DSM-5*-defined groups differed on overvaluation, cognitive ED symptoms, depression, and binge-eating frequency, the pattern of results was inconsistent and nonlinear for almost all clinical variables. Moreover, effect sizes for *DSM-5* groups were quite small, explaining a maximum of only 9% of the variance in clinical characteristics.

On balance, one reason why SEM Tree groupings and clinically significant overvaluation explained more variance in clinical characteristics than *DSM-5* groupings or single versus multiple purging methods could be measurement bias, where items/measures assessing cognitions correlate more strongly with other items/measures of cognitions, as compared to items/measures assessing behaviors. While measurement bias is a possibility, such measurement bias would have equally impacted both the SEM Tree groupings and the clinically significant overvaluation groupings. Thus, while we cannot rule out or statistically control for measurement bias, even if measurement bias is held constant, SEM Tree groupings still emerge as the stronger severity specification scheme.

Behavioral symptoms, like compensatory behaviors, have long been thought of as hallmark ED symptoms. Thus, the facts that (1) shape/weight overvaluation was superior to compensatory behavior frequency in determining SEM Tree groupings and (2) SEM Tree groups outperformed *DSM-5* severity groupings may lead some to wonder whether shape/weight overvaluation is more important to BN than compensatory behavior frequency, and whether clinicians should decrease focus on reducing compensatory behaviors. We do not draw this conclusion. Although emerging data, including our findings, suggests cognitive symptoms are more central to EDs and outperform behavioral symptoms in defining ED severity (Billman Miller et al., 2025; Forrest et al., 2022), we do not suggest neglecting treatment of behavioral symptoms. Behavioral symptoms like compensatory behaviors can have severe medical consequences (Casiero & Frishman, 2006; Mitchell, Seim, Colon, & Pomeroy, 1987) and are considered to maintain BN. Instead, we suggest that we may need to expand our conceptualization of “hallmark ED symptoms” beyond behavioral symptoms, and consider measuring and targeting shape/weight overvaluation early in treatment *in addition to* compensatory behaviors.

Although SEM Trees outperformed all other severity specification schemes, the pattern and extent to which SEM Trees captured group differences varied across clinical characteristics. SEM Tree groups differed on cognitive ED and depression symptoms, such that cognitive ED and depression symptoms increased as the level of shape/weight overvaluation increased. Anxiety symptoms followed a similar trend, although the difference between SEM Tree groups 4 and 5 was not statistically significant. Other research has also found nonlinear associations between anxiety and cognitive ED symptoms. For instance, in a sample of people with anorexia nervosa, Haynos et al. (2015) found that anxiety and cognitive ED symptoms were associated only among people with low emotion regulation difficulties. We found that binge eating frequency was similar across all SEM Tree groups, and no planned contrasts for this variable were statistically significant. Aligning with our findings, prior work in binge-eating disorder has found that binge-eating frequency is not a strong indicator of ED severity (Forrest, Smith, & Swanson, 2017; Grilo et al., 2015). Similar levels of binge eating across SEM Tree groups could be a byproduct of binge-eating frequency not being a strong correlate of ED severity.

Study strengths include a large sample comprising both adolescents and adults, strengthening the generalizability of these findings across the lifespan. Study limitations are as follows. First, all data reflect patients’ self-reported symptoms at a single timepoint. The lack of follow-up data means that we were unable to assess whether our SEM Tree groupings have predictive validity. Second, while the variables used as indicators for the latent BN severity model are evidence-based indices of severity, other unavailable variables (e.g., medical complications; Forney et al., 2016) are also important to consider when defining severity. Third, different measures of anxiety and depression were used across levels of care; we *z*-scored these measures to account for this.

Fourth, self-report measures like those used to define groups have several notable limitations. Participants could provide untruthful responses, withhold information, or be unaware of their experiences. This leads some to suggest that non-self-report metrics, such as implicit measures or physiological metrics, may be superior metrics of ED severity. However, we disagree with this conclusion, for several reasons. (1) Implicit measures have long been seen as circumventing the potential for self-report measures to be answered untruthfully. However, recent work actually suggests that self-report measures have better reliability and predictive utility than implicit measures (Corneille & Gawronski, 2024). (2) Our perception of the literature is that there is not consensus that physiological metrics are superior to self-report metrics when determining eating disorder severity. For example, weight is a relatively “physiological” metric that has for many decades been used (either formally or informally) to signify anorexia nervosa severity. However, empirical examinations of BMI as a severity metric in anorexia nervosa yield limited support for this severity operationalization (Billman Miller et al., 2025): there are many quite ill individuals with anorexia nervosa with very low BMI, yet also many quite ill individuals with anorexia nervosa with not extremely low BMI (though still underweight). (3) Assessing physiological metrics is not within the scope of practice for the vast majority of healthcare professionals who treat people with EDs (social workers, licensed professional counselors, and psychologists). (4) In many (though not all) cases, affected individuls’ reports of their experiences provide valuable information. In fact, recent work has called for the ED field to incorporate self-report measures (e.g., shape/weight overvaluation and fear of weight gain) into models to inform diagnosis and potential *DSM* revisions (Hagan & Christensen Pacella, 2024). Importantly, self-report measures are accessible and can be administered in a variety of settings, which would allow for our SEM Tree-derived severity groupings (or other schemes that may be developed or which prove to be superior) to be widely adopted.

There are also multiple sample-specific limitations. First, the outpatient sample was much smaller than residential and PHP samples, skewing our data toward those with higher severity. Second, diagnoses were assigned through unstructured clinical interviews that are specific to the clinics that provided data, and interrater reliability data are unavailable. Third, the sample was mostly White cisgender women, and data to capture socioeconomic status and education level were unfortunately unavailable. Limited sample diversity is of particular concern given evidence of bias in machine learning algorithms (Huang, Galal, Etemadi, & Vaidyanathan, 2022). Furthermore, cognitive ED symptoms may manifest differently in males and minoritized racial and ethnic groups (Bucchianeri et al., 2016), highlighting the need to validate our findings in other samples. Finally, given our use of a treatment-seeking sample, we must recognize differences between people with EDs who do versus do not seek treatment (Forrest et al., 2017). Results therefore may not be representative of all people with EDs. We must also recognize the systemic barriers that exist for many people in seeking and receiving ED treatment. These barriers disproportionately impact people belonging to marginalized groups. The exclusion of marginalized groups from ED research and treatment is a significant challenge and limitation for our field (Egbert, Hunt, Williams, Burke, & Mathis, 2022; Goel et al., 2022), where much work is needed to advance health equity.

Next steps for evaluating the utility of our SEM Tree groupings include replicating these results, comparing SEM Tree groups on relevant physiological variables, such as electrolyte imbalances (Mitchell et al., 1987) and cardiovascular consequences associated with purging (Casiero & Frishman, 2006), and assessing predictive validity. Additional research should test whether other variables or combinations of variables result in severity schemes with better validity while maximizing parsimony and clinical utility.

In sum, this study empirically defined BN severity and tested whether the empirically defined severity specification scheme outperformed existing BN severity classification schemes. We found five severity subgroups, all defined by increasing levels of shape/weight overvaluation. Our empirically defined classification scheme explained more variance in clinical characteristics than all other BN severity specification schemes, including the current *DSM-5* severity groupings. Findings suggest reconsideration of the current *DSM-5* BN severity definitions, and that (continuously modeled) overvaluation warrants consideration as a primary metric by which to define BN severity. As the ultimate goal of severity specifiers is to suggest targets for intervention, it is critical for future research to test the validity of these cutpoints in predicting BN course and treatment outcomes.

## Supporting information

Abber et al. supplementary material 1 Abber et al. supplementary material

Abber et al. supplementary material 2 Abber et al. supplementary material

## Supplementary material

The supplementary material for this article can be found at http://doi.org/10.1017/S0033291725100597.
