# Supplementary material for: Bulimia nervosa severity levels based on shape/weight overvaluation explain more variance in clinical characteristics than DSM-5 severity levels
Source: Psychol Med. 2025 Jun 30;55:e181. doi: 10.1017/S0033291725100597 (PMC12234018; doi:10.1017/S0033291725100597)
Supplement: Abber et al. supplementary material 1 — Abber et al. supplementary material [file S0033291725100597sup001.docx]

**Bulimia nervosa-specific symptoms and behaviors**

The following items on the Eating Disorder Examination–Questionnaire (EDE-Q) were used to determine whether participants reported core symptoms necessary to meet criteria for bulimia nervosa.

| Criterion | EDE-Q items | Score for exclusion |
| --- | --- | --- |
| Binge eating | (Note. Item 14 is a follow-up from item 13, which reads “Over the past 28 days, how many times have you eaten what other people would regard as an unusually large amount of food (given the circumstances)?)  14. …On how many of these times did you have a sense of having lost control over your eating (at the time that you were eating)? | 0 |
| Compensatory behaviors | 16. Over the past 28 days, how many times have you made yourself sick (vomit) as a means of controlling your shape or weight?  17. Over the past 28 days, how many times have you taken laxatives as a means of controlling your shape or weight?  18. Over the past 28 days, how many times have you exercised in a “driven” or “compulsive” way as a means of controlling your weight, shape, or amount of fat, or to burn off calories? | All items reported as 0 |
| Undue influence of shape/weight on self-esteem | 22. Has your weight influenced how you think about (judge) yourself as a person?  23. Has your shape influenced how you think about (judge) yourself as a person? | Both items scored as 0 |

**Table S1.** Comparisons of symptoms between participants admitted before vs. after the OASIS was added to the PHP and residential facilities’ intake assessment battery.

|  | Pre-OASIS admission | Post-OASIS admission |  |  |  |
| --- | --- | --- | --- | --- | --- |
|  | *M* (*SD*) | *M* (*SD*) | *t* | *p* | Cohen’s *d* |
| EDE-Q Global | 4.41 (1.19) | 4.27 (1.16) | 1.71 | .09 | -0.12 |
| Overvaluation | 5.08 (1.45) | 5.14 (1.36) | -0.59 | .56 | 0.04 |
| Depression score | 39.45 (11.36) | 36.97 (11.86) | 3.07 | .002 | -0.21 |
| Compensatory behavior frequency | 8.33 (5.99) | 6.49 (5.12) | 4.67 | < .001 | -0.34 |

**Table S2.** Comparisons of non-normally distributed data (log-transformed variables and non-parametric tests) for SEM Tree groups

|  | 1  *n* = 34 | 2  *n* = 152 | 3  *n =* 65 | 4  *n* = 177 | 5  *n* = 582 |  |  |  |
| --- | --- | --- | --- | --- | --- | --- | --- | --- |
|  | *Median* or *M* (*SD*) | *Median* or *M* (*SD*) | *Median* or *M* (*SD*) | *Median* or *M* (*SD*) | *Median* or *M* (*SD*) | *F* or *H* | *p* | $\eta$^2^ |
| Binge eating (nonparametric) | 2.75 | 2.50 | 3.75 | 3.25 | 3.75 | 19.51 | < .001 | 0.02 |
| Binge eating (log transformed) | 1.21 (0.77) | 1.17 (0.71) | 1.48 (0.72) | 1.32 (0.77) | 1.44 (0.75) | 4.51 | .002 | 0.02 |
|  | 1 vs. 2+3+4+5 | | 2 vs. 3+4+5 | | 3 vs. 4+5 | | 4 vs. 5 | |
|  | *t* | *p* | *t* | *p* | *t* | *p* | *t* | *p* |
| Binge eating (nonparametric) | 0.95 (37) | .80 | 3.23 (37) | .01 | -0.93 (37) | .81 | 2.06 (37) | .17 |
| Binge eating (log transformed) | 1.10 (36.26) | .28 | 3.27 (244.18) | .001 | -1.13 (83.60) | .26 | 1.79 (276.98) | .08 |

*Note. Medians* are presented for nonparametric tests; *means* and *standard deviations* are presented for log-transformed variables.

**Table S3.** Gender, race, and age distributions for DSM-5 groups

|  | Mild  *n* = 462 | Moderate  *n* = 182 | Severe  *n =* 226 | Extreme  *n* = 139 |  |  |
| --- | --- | --- | --- | --- | --- | --- |
|  | *n* (%) | *n* (%) | *n* (%) | *n* (%) | $\chi$^2^ | *p* |
| Gender |  |  |  |  | 6.53 | .67 |
| Cis man | 3 (0.7) | 0 (0) | 0 (0) | 0 (0) |  |  |
| Cis woman | 456 (98.7) | 181 (99.5) | 226 (100) | 139 (100) |  |  |
| Trans man | 2 (0.4) | 1 (0.6) | 0 (0) | 0 (0) |  |  |
| Trans woman | 0 (0) | 0 (0) | 0 (0) | 0 (0) |  |  |
| Gender diverse | 1 (0.2) | 0 (0) | 0 (0) | 0 (0) |  |  |
| Race |  |  |  |  | 11.79 | .86 |
| American Indian or Alaska Native | 4 (0.9) | 2 (1.1) | 1 (0.5) | 1 (0.7) |  |  |
| Asian | 17 (3.8) | 9 (5.0) | 8 (3.6) | 3 (2.2) |  |  |
| Black | 13 (2.9) | 7 (3.9) | 6 (2.7) | 4 (3.0) |  |  |
| Hispanic | 38 (8.4) | 9 (5.0) | 16 (7.3) | 8 (5.9) |  |  |
| Multiracial | 33 (7.3) | 15 (8.3) | 15 (6.8) | 3 (2.2) |  |  |
| White | 343 (76.1) | 136 (75.6) | 172 (78.2) | 115 (85.2) |  |  |
| Not reported | 3 (0.7) | 2 (1.1) | 2 (0.9) | 1 (0.7) |  |  |
|  | *M* (*SD*) | *M* (*SD*) | *M* (*SD*) | *M* (*SD*) | *F* | *p* |
| Age | 24.5 (9.3) | 25.2 (8.9) | 24.2 (8.8) | 25.8 (10.0) | 1.16 | .32 |

**Table S4.** Comparisons of non-normally distributed data (log-transformed variables and non-parametric tests) for DSM-5 groups

|  | Mild  *n* = 462 | Moderate  *n* = 182 | Severe  *n* = 226 | Extreme  *n* = 139 |  |  |  |
| --- | --- | --- | --- | --- | --- | --- | --- |
|  | *n* (%) or *M* (*SD*) | *n* (%) or *M* (*SD*) | *n* (%) or *M* (*SD*) | *n* (%) or *M* (*SD*) | $\chi$^2^ or *F* | *p* | $\eta$^2^ |
| Binge eating (nonparametric) | 2.50 | 3.75 | 3.75 | 6.25 | 52.20 | < .001 | 0.05 |
| Binge eating (log transformed) | 1.22 (0.75) | 1.38 (0.63) | 1.43 (0.70) | 1.75 (0.83) | 14.27 | < .001 | 0.05 |
|  | Mild vs. Moderate + Severe +  Extreme | | Moderate vs. Severe + Extreme | | Severe vs. Extreme | |  |
|  | *t* or *W* | *p* | *t* or *W* | *p* | *t* or *W* | *p* |  |
| Binge eating (nonparametric) | 5.52 | < .001 | 3.63 | .001 | 4.01 | < .001 |  |
| Binge eating (log transformed) | 6.05 (863.37) | < .001 | 2.57 (394.04) | .01 | 3.13 (267.21) | .002 |  |

**Table S5.** Gender, race, and age distributions for dichotomous shape/weight overvaluation groups and multiple purging groups.

|  | Overvaluation < 4  *n* = 143 | Overvaluation >= 4  *n* = 870 |  |  | Single purging  *n* = 776 | Multiple purging  *n* = 233 |  |  |
| --- | --- | --- | --- | --- | --- | --- | --- | --- |
|  | *n* (%) | *n* (%) | $\chi$^2^ | *p* | *n* (%) | *n* (%) | $\chi$^2^ | *p* |
| Gender |  |  | 7.50 | .06 |  |  | 2.12 | .55 |
| Cis man | 2 (1.4) | 1 (0.1) |  |  | 3 (0.4) | 0 (0) |  |  |
| Cis woman | 141 (98.6) | 865 (99.4) |  |  | 769 (99.1) | 233 (100) |  |  |
| Trans man | 0 (0) | 3 (0.3) |  |  | 3 (0.4) | 0 (0) |  |  |
| Trans woman | 0 (0) | 0 (0) |  |  | 0 (0) | 0 (0) |  |  |
| Gender diverse | 0 (0) | 1 (0.1) |  |  | 1 (0.1) | 0 (0) |  |  |
| Race |  |  | 9.04 | .17 |  |  | 9.76 | .14 |
| American Indian or Alaska Native | 2 (1.5) | 6 (0.7) |  |  | 4 (0.5) | 4 (1.7) |  |  |
| Asian | 10 (7.4) | 26 (3.0) |  |  | 32 (4.2) | 5 (2.2) |  |  |
| Black | 3 (2.2) | 27 (3.2) |  |  | 19 (2.5) | 11 (4.8) |  |  |
| Hispanic | 6 (4.4) | 65 (7.6) |  |  | 58 (7.7) | 13 (5.6) |  |  |
| Multiracial | 10 (7.4) | 56 (6.6) |  |  | 53 (7.0) | 13 (5.6) |  |  |
| White | 104 (76.5) | 667 (78.1) |  |  | 583 (77.2) | 183 (79.2) |  |  |
| Not reported | 1 (0.7) | 7 (0.8) |  |  | 6 (0.8) | 2 (0.9) |  |  |
|  | *M* (*SD*) | *M* (*SD*) | *F* | *p* | *M* (*SD*) | *M* (*SD*) | *F* | *p* |
| Age | 23.5 (9.6) | 24.6 (9.2) | 0.71 | .40 | 24.6 (9.2) | 25.0 (9.4) | 0.30 | .59 |

**Table S6.** Comparisons of non-normally distributed data (log-transformed variables and non-parametric tests) for dichotomous shape/weight overvaluation groups and single vs. multiple purging groups

|  | Overvaluation < 4  *n* = 143 | Overvaluation >= 4  *n* = 870 |  |  |  |
| --- | --- | --- | --- | --- | --- |
|  | *Median* or *M* (*SD*) | *Median* or *M* (*SD*) | *t* or *W* | *p* | *r* or *d* |
| Eating disorder symptoms (nonparametric) | 2.50 | 6.00 | 8324.50 | < .001 | .52 |
| Eating disorder symptoms (log transformed) | 0.78 (0.45) | 1.43 (0.19) | -17.21 | < .001 | 2.69 |
|  | Single purging  *n* = 776 | Multiple purging  *n* = 233 |  |  |  |
|  | *Median* or *M* (*SD*) | *Median* or *M* (*SD*) | *t* or *W* | *p* | *r* or *d* |
| Overvaluation (nonparametric) | 6.00 | 6.00 | 75236 | < .001 | .13 |
| Overvaluation (log transformed) | 1.75 (0.35) | 1.84 (0.25) | 4.52 | < .001 | 0.28 |
| Eating disorder symptoms (nonparametric) | 0.11 | 0.53 | 67481 | < .001 | .19 |
| Eating disorder symptoms (log transformed) | 1.31 (0.35) | 1.44 (0.25) | 6.39 | < .001 | 0.40 |

*Note.* Eating disorder symptoms are z-scored. *Medians* are presented for nonparametric tests; *means* and *standard deviations* are presented for log-transformed variables. For nonparametric tests, *r* values are presented as a measure of effect size. For log-transformed variables where t tests were performed, Cohen’s *d* values are presented as a measure of effect size.
