# Supplementary material for: Bulimia nervosa severity levels based on shape/weight overvaluation explain more variance in clinical characteristics than DSM-5 severity levels
Source: Psychol Med. 2025 Jun 30;55:e181. doi: 10.1017/S0033291725100597 (PMC12234018; doi:10.1017/S0033291725100597)
Supplement: Abber et al. supplementary material 2 — Abber et al. supplementary material [file S0033291725100597sup002.docx]

#---------------BN SEM Trees---------------#

#0. load packages

library(foreign)

library(haven)

library(OpenMx)

library(lavaan)

library(devtools)

devtools::install_github("brandmaier/semtree", force = TRUE)

#read in BN_fiml data; this dataframe includes only five variables: the three indicators of the outcome model (ede, dep, and anx) and the two covariates (sw_overval and compens)

BN_observed=colnames(BN_fiml[,c(1,2,3)])

BN_latent=c("f1")

BN_cfa <- mxModel("Model", type="RAM",

manifestVars=c(BN_observed),

latentVars=c("f1"),

#factor loadings

mxPath(from="f1", to=c("ede","dep","anx"),

free=c(F,F,F),values=c(1,1,1),labels=c("l1","l2","l3")),

#manifest means

mxPath(from = 'one', to = BN_observed,values=2,labels=paste(paste("m"), 1:3,sep="")),

#residual variBNces

mxPath(from=BN_observed, arrows=2,labels=paste(paste("resid"), 1:3,sep=""),lbound=0),

#latent mean

#mxPath(from = 'one', to = "f1",values=1,free=F),

#latent variance

mxPath(from=BN_latent, arrows=2,free=F,values=1,labels=c("v1")),

#data

mxData(BN_fiml[,c(1,2,3)],type="raw")

)

BN_cfa.out = mxRun(BN_cfa)

summary(BN_cfa.out)

#Open Mx tree

my.control = semtree.control(method="fair", seed=1, min.N=40, missing = "party")

BN_tree <- semtree(model = BN_cfa.out, data = BN_fiml, control = my.control)

pdf("BN_tree.pdf")

plot(BN_tree)

dev.off()

#random forest--mtry 1

BN_semforest.out <- semforest(BN_cfa.out,BN_fiml,control)

set.seed(436)

BN_vim.forest1 <- varimp(BN_semforest.out)

pdf("BN_varimp1_edit.pdf")

plot(BN_vim.forest1, cex.axis = 2.1, xlab = "-2 log likelihood", cex.lab = 2.1)

dev.off()

vimp_means <- as.data.frame(BN_vim.forest1[["importance"]])

mean(vimp_means$sw_overval, na.rm = TRUE)

mean(vimp_means$compens, na.rm = TRUE)

#random forest--mtry 2

BN_semforest.out2 <- semforest(BN_cfa.out,BN_fiml,control2)

set.seed(436)

BN_vim.forest2 <- varimp(BN_semforest.out2)

pdf("BN_varimp2.pdf")

plot(BN_vim.forest2)

dev.off()
